# Low HIV-risk aligned discontinuation among HIV pre-exposure prophylaxis users within public HIV clinics in Kenya: A mixed method study

**DOI:** 10.1371/journal.pgph.0004493

**Published:** 2025-04-28

**Authors:** Njeri Wairimu, Kenneth Ngure, Vallery Ogello, Emmah Owidi, Paul Mwangi, Lydia Etyang, Winnie Waituika, Margaret Mwangi, Dominic M. Githuku, Simon Maina, Elizabeth Irungu, Nelly Mugo, Kenneth K. Mugwanya

**Affiliations:** 1 Partners in Health Research and Development, Centre for Clinical Research, Kenya Medical Research Institute, Nairobi, Kenya; 2 School of Public Health, Jomo Kenyatta University of Agriculture and Technology, Nairobi, Kenya; 3 Jhpiego, Nairobi, Kenya; 4 Kenya Medical Research Institute, Nairobi, Kenya; 5 Department of Global Health, University of Washington, Seattle, Washington, United States of America; 6 Department of Epidemiology, University Washington, Seattle, Washington, United States of America; Weill Cornell Medicine, UNITED STATES OF AMERICA

## Abstract

Adherence to oral HIV pre-exposure prophylaxis (PrEP) is crucial for its effectiveness, however, studies have shown that PrEP use wanes within the first six months. We sought to understand reasons for discontinuation among individuals previously accessing PrEP from HIV clinics. Between November 2020 – January 2023, we conducted a mixed methods sub-study within a programmatic study to improve the efficiency of PrEP delivery in four public HIV clinics in Kenya (ClinicalTrials.gov number NCT04424524). We used random simple stratification to select individuals who had discontinued PrEP and completed surveys; we purposively sampled a subset of participants for in-depth interviews. Quantitative data were analyzed descriptively; qualitative data were analyzed thematically guided by the socio-ecological model. Overall, 300 participants completed surveys; median age was 35 years (interquartile range 28-43), 61% were female and 57% were married/cohabiting. Majority (76%) discontinued PrEP because of low perceived risk of HIV acquisition. Nearly half (43.7%) reported not being at risk, 23% had separated from their partners or had partners who were virally suppressed (6%), 3.3% were discontinued by healthcare providers. Other reasons for discontinuation were PrEP use concerns (15.6%) including concerns about side effects (8.7%) and daily pill burden (6%). Accessibility challenges (4%), and opportunity costs such as fear of missing/losing work (1%) were reported less frequently. Similarly in qualitative interviews, participants (n=30) reported PrEP discontinuation was mainly driven by perceived low HIV risk due to changes in relationship dynamics (separation/partner relocation), partner achieving viral suppression for those in serodifferent partnerships and reduced sexual activity (individual and interpersonal factors). Other themes included perceived HIV/PrEP stigma (community factors), frequency of clinic visits and long wait times (structural/institutional factors). PrEP discontinuation was mainly associated with perceived low HIV risk in this study population. Prevention-effective adherence counselling is essential in supporting individuals to correctly assess HIV risk to inform appropriate discontinuation.

## Introduction

Oral HIV pre-exposure prophylaxis (PrEP) effectiveness is highly dependent on sustained user adherence and persistence [1–3]. On average, PrEP continuation was 65% one month post-initiation, based on data gathered from clinical trials, demonstration projects and implementation programmes conducted in various countries [4]. Despite the gradual increase in PrEP implementation in many sub-Saharan Africa countries, early discontinuation and non-persistence rates remain frequent [5–7]. For example, the Determined, Resilient, Empowered, AIDS-free, Mentored and Safe (DREAMS) program, recorded only 37% PrEP continuation three months post-initiation among adolescent girls and young women [8]. Similarly, the Partners Scale-Up Project in Kenya reported high rates of PrEP discontinuation, with only 57% and 23% of participants returning to the clinic for PrEP refills at one and 12 months respectively [9]. In a systematic review of PrEP discontinuation across different regions and populations, sub-Sahara Africa recorded the highest discontinuation rates, with 48% discontinuing within six months, compared to 9% in South America. The review also found that cisgender girls and women had a higher rate of discontinuation (43%) than gay and bisexual men who have sex with men (32%) [5]. These rates of discontinuation highlight the importance of understanding client reasons for PrEP discontinuation to inform adherence messaging.

In Kenya, PrEP is delivered within HIV care clinics after demonstration projects showed high feasibility, and success of its integration into the existing HIV delivery systems [10–14]. Public HIV clinics employ various strategies to follow-up PrEP clients who fail to return on their scheduled date including adherence counselling, support groups, phone calls, and text message reminders [15,16]. Despite these efforts to retain individuals on PrEP, continuation challenges remain high across different populations of PrEP users. Reasons for PrEP discontinuation among trial participants have been shown to include stigma, daily pill burden, side effects concerns, opposition from sexual partners, inadequate social support, and logistical barriers (e.g., distance and busy work schedules) [17–20].

PrEP discontinuation has also been attributed to individuals’ perceptions of HIV risk, which can fluctuate over time. Studies have found that decisions to initiate PrEP are often driven by perceived high HIV risk, due to factors such as condomless sex, multiple sexual partnerships, unknown partner HIV status and engaging in transactional sex. As seasons of risk change, individuals may reassess the need for PrEP, particularly when sexual behaviors associated with HIV risk change – such as infrequent sexual activity or having a partner living with HIV with suppressed viral load – motivating discontinuation [20–23].

However, there is paucity of data on PrEP discontinuation among individuals accessing PrEP from public HIV clinics, which may differ from research setting findings. Understanding these factors is important as public HIV clinics in Kenya are the primary point for PrEP delivery, and addressing PrEP discontinuation could improve retention strategies and support Kenya’s goal to end the AIDS epidemic by 2030 [24]. We sought to understand the reasons for PrEP discontinuation among individuals who discontinued PrEP in four public HIV clinics in central Kenya.

## Materials and methods

### Study design and participants

Between 27 November 2020 to 11 January 2023, we conducted a convergent parallel, mixed methods sub-study nested within The Efficiency Study (ClinicalTrials.gov: NCT04424524), a programmatic implementation project to evaluate the feasibility and acceptability of a differentiated direct-to-pharmacy PrEP refill visits to improve the efficiency of PrEP delivery at four public HIV clinics in central Kenya [25]. The parent study was powered for the primary outcomes of patient waiting and contact time with healthcare providers. With a sample size of 500 overall, we had >80% power to demonstrate >75% reduction (i.e., a reduction from ~90 to <30 minutes) in time spent in the clinics by clients with providers. Specifically, the sub-study on discontinuation was planned to be descriptive rather than hypothesis testing. Thus, the focus was not power but the precision in estimates around the proportion of reported key reasons for discontinuation. We estimated that the sample size of 300 participants who meet the eligibility for the PrEP discontinuation survey (which >60% (300/500) of parent study), was sufficient to provide high precision (i.e., tight confidence limits) in estimates for the proportion of programmatically meaningful reported reasons for discontinuation or disengagement in PrEP care. Participants enrolled in both the parent study and sub-study were individuals who had received PrEP from any of the four clinics and were ≥18 years.

Among participants enrolled in The Efficiency Study, we generated a list of individuals who initiated but later discontinued PrEP, based on the following eligibility criteria: 1) started PrEP but did not return to the clinic for a refill within 6 months; 2) started PrEP and returned to the clinic for one refill after PrEP initiation but did not return to the clinic for another refill within the next 6 months; and, 3) started PrEP, returned to the clinic for at least two refills after PrEP initiation but did not return to the clinic for another refill within the next 6 months. We contacted all individuals who had discontinued PrEP until we found those who were reachable by phone and agreed to participate in the study. Participants who did not meet the PrEP discontinuation criteria or were not accessing PrEP from any of the four clinics were not included.

Participants were initially contacted via phone by healthcare providers who briefly introduced the study and research team. Trained research staff contacted participants who gave verbal consent for their contact details to be shared with them, to describe the research and offer them an opportunity to participate and schedule surveys to understand their reasons for discontinuing PrEP. A subset of these participants was selected to participate in-depth interviews to gain deeper insight into their reasons for PrEP discontinuation. We used purposive sampling and stratified participants by gender. We contacted participants who expressed interest and availability for interview participation, and continued this process until we had equal representation of male and female participants.

### Data collection

Research assistants administered brief surveys (S1 Appendix) via phone to gather participants’ demographic data and their reasons for PrEP discontinuation. Data was collected in real time and uploaded to Research Electronic Data Capture (REDCap), a web-based application [26]. Qualitative data was collected by trained social scientists using semi-structured interview guides (S2 Appendix) to solicit information related to PrEP discontinuation including: HIV risk perceptions (e.g., motivation to start and stop PrEP use), general PrEP experiences and perceptions (e.g., side effects, pill burden), HIV clinic experiences (e.g., waiting times, frequency of visits), and stigma. Interviews were conducted in a private room in the facilities or over the phone in participants’ preferred language (English or Kiswahili), audio recorded and simultaneously translated and transcribed verbatim into English. Prior to the interviews, participants were reminded to be in a private location where they would feel comfortable discussing PrEP and HIV related topics to ensure confidentiality during phone data collection. On the day of the interview, interviewers confirmed that participants were in a private space before beginning data collection.

Interviews took an average of 52 minutes and quality checks were performed on all transcripts. This involved cross-checking the accuracy of each transcript against the respective audios, reviewing unclear sections by the interviewer or second member of the team, and ensuring completeness and consistency in formatting. Surveys and interview guides were developed by the study team in English and pilot-tested prior to data collection, with feedback from the pilot used to refine the tools. During data collection, study staff, proficient in local languages (Kiswahili), translated questions into participants’ preferred language to ensure clarity and accurate understanding.

### Data analysis

For quantitative data, we used descriptive statistics to present participant demographics and analyze reasons for PrEP discontinuation. For continuous variables like age, we reported the median and interquartile range (IQR), and proportion for the categorical variables such as sex. All analyses were conducted using R statistical software (version 4.3.1, R Foundation for Statistical Computing, Vienna, Austria).

For qualitative data, authors NW, EO, and VO, all trained in qualitative methodology, analyzed qualitative data using Dedoose software, version 9.0.107 (SocioCultural Research Consultants, LLC, Los Angeles, USA). A thematic codebook was developed using deductive approaches based on the interview guide, and inductive approaches based on themes emerging from transcripts [27,28]. The coding team tested and refined the codebook by independently coding the same three randomly selected transcripts to ensure intercoder reliability. The team held weekly meetings to discuss any disagreements, which were resolved through discussions until consensus was reached. The remaining transcripts were distributed equally and each coded by one person. We used the socio-ecological model (SEM) to guide the analysis and understand participants’ decisions to discontinue PrEP use. The SEM has been widely used in public health, as it recognizes that health behavior, such as PrEP use, is more than a result of individuals’ actions and decisions, but is also influenced by the environment in which they exist [29,30]. It argues that individuals’ behaviors are shaped by various factors including: individual, interpersonal, community and structural/institutional factors. We used this model to thematically analyze how these factors influenced PrEP discontinuation. We followed the Consolidated Criteria for Reporting Qualitative Research guidelines in our qualitative methods [31]. Simultaneous integration of qualitative and quantitative data approaches was achieved by identifying concepts that provided meaning to the quantitative results.

### Ethical approval

This study was approved by the Scientific and Ethics Review Unit at the Kenya Medical Research Institute (KEMRI), Kenya, and the Human Subjects Division at the University of Washington (UW), USA. In this study, participants were verbally consented using an oral consent guide (S3 Appendix) as they had discontinued PrEP and stopped attending facility visits. Oral consent was documented on a consent log (S4 Appendix) by study staff. The oral consent guide was reviewed and approved by the KEMRI and UW institutional review boards. All participants were reimbursed 500 Kenyan shillings for their time and effort after for completing either a survey or an interview.

### Inclusivity in global research

Additional information regarding the ethical, cultural, and scientific considerations specific to inclusivity in global research is included in the Supporting Information (S1 Checklist).

## Results

### Participant characteristics

A total of 300 participants completed brief surveys; median age was 35 years (IQR 23-43), 61% were female, 57% were either married or cohabiting with a partner, and 41% were in HIV serodifferent relationships. Majority (77%) of participants were aware of the HIV status of their last sexual partner and slightly more than half (55%) had used PrEP for more than six months before discontinuing (Table 1).

**Table 1:** Demographic characteristics at PrEP Discontinuation (n=300).

Characteristic	n (%) Median [IQR]
**Age (years)**	
Median [Min, Max]	35.0 [28.0, 43.0]
< 25 years	35 (11.7%)
≥ 25 years	265 (88.3%)
**Sex**	
Male	117 (39.0%)
Female	183 (61.0%)
**Marital status**	
Never married	39 (13.0%)
Cohabiting/married	171 (57.0%)
Separated/divorced/widowed	90 (30.0%)
**Population type (*M=2*)**	
General population	138 (46.0%)
Serodiscordant couple	124 (41.3%)
Female sex workers	7 (2.3%)
Men who have sex with men	2 (0.6%)
Unknown/No entry	27 (9.0%)
**Education levels (*M=1*)**	
Primary education and below	74 (24.7%)
Secondary education	156 (52.0%)
Post-secondary education	69 (23.0%)
**Income per month**	
No income	45 (15.0%)
<= 10,000 Ksh	79 (26.3%)
> 10,000 Ksh	150 (50.0%)
Declined to answer	26 (8.7%)
**Duration on PrEP before discontinuation**	
≤ six months	136 (45.3%)
> six months	164 (54.7%)
**HIV status of the last person with whom you had sex with**	
HIV negative	114 (38.0%)
HIV positive	116 (38.7%)
Unknown	70 (23.3%)

***M*** represents missing values.

### Main reasons for PrEP discontinuation

Table 2 summarizes the key reasons for PrEP discontinuation. Majority of the 300 participants who discontinued PrEP perceived themselves to be at low risk of HIV acquisition (76%); nearly half (44%) selected the general response *“felt I was no longer at risk for HIV”* as their main reason for PrEP discontinuation. Some participants (23%) perceived themselves no longer at risk because they had separated from their sexual partner or their partner living with HIV was on treatment and had achieved viral suppression (6%), with only a small proportion (3%) of participants being discontinued by healthcare providers due to low HIV risk. A few participants (16%) reported concerns about PrEP medication, specifically, 9% reported experiencing or having concerns about side effects and 6% reported daily pill burden. Other less frequently reported reasons for PrEP discontinuation included challenges with accessing the clinic (4%), and opportunity costs such as fear of missing/losing work (1%).

**Table 2:** Reasons for PrEP discontinuation (n=300).

Reasons for PrEP discontinuation	n (%)
Perceived Low Risk of HIV (n = 229)	
Felt I was no longer at risk for HIV	131 (43.7%)
Separated – no longer at risk	69 (23.0%)
HIV+ partner on ART and virally suppressed	18 (6.0%)
Clinician initiated PrEP stop	10 (3.3%)
Stopped after conceiving	1 (0.3%)
Concerns about PrEP Use (n = 47)	
Had or was concerned about side effects	26 (8.7%)
Concerned about too many pills to take every day	18 (6.0%)
PrEP is not the right HIV prevention method for me	3 (1.0%)
Logistical and Social Barriers (n = 24)	
Concerned about transport to and from the clinic	12 (4.0%)
Concerned about missing work or losing work	4 (1.3%)
Did not have enough information about using PrEP	3 (1.0%)
Other	3 (1.0%)
Concerned about family finding out I am taking PrEP	1 (0.3%)
Wait time at clinic was too long	1 (0.3%)

### Qualitative findings

**Fig 1:**
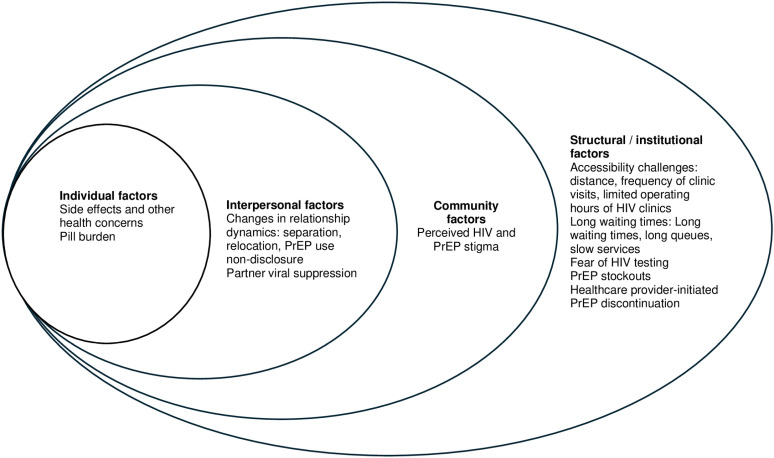
Figure of the socio-ecological model illustrating multi-level factors contributing to PrEP discontinuation.

Participants who completed in-depth interviews (n=30) had a median age of 39.5 years (IQR 29-45.5) and half were female. The median duration of PrEP use before discontinuation was 21 months (IQR 5.25-40.5), and majority (17/30) reported being in serodifferent relationship (Table 3). The primary reasons for PrEP discontinuation aligned with perceived low HIV risk states and included: viral suppression, separation, perceived changes in sexual behaviour, or partner relocation (individual and interpersonal factors). Other factors that influenced PrEP discontinuation included PrEP use concerns such as side effects and pill burden (individual factors), PrEP stigma (community factors), frequency of clinic visits, and long wait times (structural/institutional factors). We describe these factors that influenced PrEP discontinuation at each level of the socio-ecological model: individual, interpersonal, community, and structural/institutional levels (Fig 1).

**Table 3:** Demographic characteristics of qualitative participants.

		Overall (N=30)
**Sex**		
	Female	15 (50.0%)
	Male	15 (50.0%)
**Age**		
	Mean (SD)	38.7 (11.6)
	Median [IQR]	39.5 [29.0,45.5]
**Relationship Type**		
	HIV-serodiscordant	17 (56.7%)
	Married	2 (6.7%)
	Separated	2 (6.7%)
	Single	5 (16.7%)
	Unknown partner status	3 (10.0%)
	Widow	1 (3.3%)
**Duration on PrEP**		
	Mean (SD)	23.7 (19.9)
	Median [IQR]	21.0 [5.25,40.5]

## Individual and interpersonal factors

### Changes in relationship dynamics

Participants discontinued PrEP when they felt no longer at risk of HIV acquisition. Low HIV risk perception was associated with changes in relationship dynamics following separation from sexual partners living with HIV and were the main reason for PrEP use.

*If I had continued to live with that man, I would be taking them (PrEP) until now. If he would have changed and stopped that habit of his, of staying with girls (having other sexual partners) ... So, I felt that I should just leave him and also leave those drugs … When I broke up with that man, I stopped everything … As in swallowing [PrEP], he was the one who was making me take them. I had the fear that he would bring to me AIDS, and I would die and leave my baby. (28-year-old female, unknown partner HIV status)*

Some participants perceived themselves to be at low HIV risk following partner relocation which led to reduced sexual activity when their partners were away for long durations. This geographic separation influenced their decision to stop using PrEP, noting that they were not at an on-going risk for HIV, with some opting to use alternative methods of HIV prevention such as condoms when they reunited with their partners.

*At the moment, I am not using PrEP since right now my wife is not here. I don’t live with her here; she is upcountry with the kids. So, I saw it best to leave PrEP, because there is no one am engaging with [sexually] … but when I go home in the period that I have not been using PrEP, I usually use condoms. (34-year-old male, serodifferent relationship)*

Additionally, some participants discontinued PrEP use as they had not disclosed it to their partners, fearing conflict if their partners found out and perceived them to be living with HIV or have other sexual partners. One participant discontinued PrEP use as a result of her view of morality. The participant, a widow, explained that she had stopped being sexually active because she believed that it was not right for her to continue engaging in sex at her age and no longer needed PrEP as a result.

*…and then I just felt like I should stop it (PrEP) because actually I am not very [sexually] active … I’m now at the age of 58 I’m going 59, my husband died in 2012 and actually I’m not a fan of that (engaging in sex) … I just felt I am not doing the right thing at my age that is because of my status. (58-year-old widow)*

### Partner viral suppression

Participants whose partners were living with HIV reported being counselled by healthcare providers on the importance of adherence to HIV treatment in achieving viral suppression as a method of reducing the risk of HIV transmission. They reported supporting their partners with adherence to HIV medication while they were on PrEP themselves, and discontinuing PrEP when their partners’ viral load was undetectable as they were confident that they were protected from HIV acquisition based on the counselling they had received.

*I’m not taking PrEP now but after we had come to an agreement, and he takes the drugs very nicely, I monitor him, and the viral load was undetectable. They (healthcare providers) told me … if your partner takes the drugs very well that the viral load is undetectable, because U=U (undetectable is equal to untransmissible), I think that is what I was told. Because as we are speaking now it is undetectable. (30-year-old female, serodifferent relationship)*

### Side effects and other health concerns

Participants reported experiencing side effects associated with PrEP use such as vomiting, dizziness, appetite loss, headaches, nausea, heart palpitations, skin rashes, and general malaise and were concerned that some symptoms had persisted beyond the time advised by their healthcare providers during counseling. Some participants however reported that even though they did not experience any PrEP side effects, they were concerned about the potential side effects of prolonged PrEP use such as cancer and kidney disease.

*I was just feeling like vomiting, and also feeling dizziness … When you take them and you feel that you don’t have an appetite … I don’t know what I was feeling, I felt that I should just leave them alone so I can see whether I will stop feeling like that. And I was told that if I take them for one month it (side effects) will stop, and it did not stop. (31-year-old female, unknown partners HIV status)*

*[I was concerned about] staying with medication every day in the body, you know you ask yourself that this medication that you take every day in your body don’t they have any side effects? I used to ask myself such questions and I felt like no I will not be taking the medication daily and I am not sick. (44-year-old female, single-previously in serodifferent relationship)*

One participant reported provider-initiated PrEP discontinuation after experiencing severe PrEP side effects, that is, kidney disease, which persisted even after treatment, and eventually led to hospital admission.

*Health-wise, it was not good to me because it was my first time to start taking it (PrEP) and I noticed that there were some patches growing on my skin and after sometimes I developed kidney challenges. So, I went back to the hospital where I used to pick them and they gave me drugs. After three weeks or so, the same problem came up again to a point I was admitted to the hospital and that is when I stopped [taking PrEP]. (25-year-old male, single-previously in serodifferent relationship)*

Other reported health concerns were perceived contraindications of PrEP use alongside other medication. Participants were specifically worried about the effect of using different medications at the same time on their health and felt safer taking medication prescribed for similar health conditions together before introducing more.

*…I was given medication to treat pneumonia. So, I felt that the drugs were so many, so I decided to use the pneumonia drugs plus the COVID vaccine, [which] I felt that the vaccine was powerful so that the body doesn’t become overloaded with chemicals … so I decided to leave PrEP and concentrate in the Corona vaccine so it doesn’t affect me much since taking both would have side effects. This is something I thought by myself the doctor did not tell me, and I decided to stay with the Corona vaccine and see how my body reacts. (47-year-old male in serodifferent relationship)*

### Pill burden

Pill burden also contributed to PrEP discontinuation among some participants who had challenges taking PrEP due to the size and taste of the pill, which they described as too big and bitter to swallow, with some reporting that the daily, same-time dosing regimen was too restrictive. Adherence challenges were also caused by forgetting and logistical challenges like working late or travelling.

*That [taking PrEP every day] contributed [to me stopping PrEP], because sometimes I would leave, and I was not there when I needed to take them. You see, or other times I am far away, and I was required at that time to take them. Another reason is that it was a must to take PrEP at a certain time; you can’t delay because time is running out. All these used to frustrate me. (42-year-old male in serodifferent relationship)*

## Community factors

### Perceived HIV and PrEP stigma

Participants were uncomfortable accessing PrEP from the same clinic as people living with HIV for fear of being judged as living with HIV themselves. Some participants discontinued PrEP because they were worried about the potential stigma they would face in the community from frequently visiting the hospital and queuing at HIV clinics. There were also concerns about inadvertent PrEP use disclosure if their pills were seen or their sound against the pill bottles heard by other people.

*…I even saw that these drugs were good and that is why I used them. When going there (HIV clinic) for refills, I used to find a lot of people and told myself that, “these people will start saying that I am sick (living with HIV) and that is why I go for my drugs this other side (HIV clinic).” And that is why I had to stop. Because if I come here and stay for such long hours, people will start wondering what I came for here. (42-year-old female, separated)*

One participant reported that her using PrEP caused stress to her son and strained their relationship. When he saw the PrEP pills and looked them up, he made the conclusion that she was living with HIV and resorted to alcohol use, leading her to the decision to stop using PrEP.

*…I have a son who is in his 20s then he went and searched what the medicine [PrEP] were online and since that day he is not okay he disturbs me, he takes alcohol and he still young not yet even 25yrs so he tells me when he is drunk that there is a day I went and saw those drugs … you see maybe he got shocked that the mother may have gotten infected with HIV and concluded that they are just ARVs you see, so I just felt that now I don’t want them [PrEP]. (44-year-old female, single-previously in serodifferent relationship)*

## Structural/institutional factors

### Accessibility challenges and long waiting times

Participants highlighted various challenges related to access and convenience that led to PrEP discontinuation including long distances to HIV clinics posing logistical challenges such as travel costs and time constraints. They also expressed concerns about the frequency of clinic visits for PrEP refills, limited operating hours of HIV clinics, long waiting times from long queues, and slow services while accessing PrEP, with some adding that it affected their jobs as they had limited time off work.

*[After I stopped using PrEP] in one way or the other I felt that I was relieved the burden of travelling since I was travelling from far … because you find that if it is something you pick close by, or something you can pick without struggling, then it’s hard to stop. For example, I am in [location redacted], imagine if I was supposed to pick them today or tomorrow, you see that I could have struggled. (69-year-old male in serodifferent relationship)*

*Time also contributed. When you go to the clinic, you find other people waiting. Some have big problems and end up staying with the doctor for long, so you end up staying at the hospital for half a day … I used to ask myself whether I can go there and get served faster for me to go back to work or I just go to another clinic. You will end up staying. It was a lot of time … At times you get there and there is no doctor and when he comes, he walks away. (49-year-old female in serodifferent relationship)*

Other structural factors that led to PrEP discontinuation included the fear of HIV testing at every PrEP refill visit, PrEP stockouts, and healthcare provider-initiated PrEP discontinuation with no reason given. One participant recounted negative experiences with healthcare providers who scolded clients who had poor PrEP adherence:

*But there is another madam (healthcare provider) that shouts at us when giving us those drugs, so, I felt I should leave them alone. I felt that I should resume using condoms. When reminding us, they shout at us when we are even in a market, you are among many people. They ask you, “Now you, you forgot to go for your drugs?” I thought that I should just leave them because she might embarrass you, that one. You should call someone aside and you talk, so I saw that I should just leave them. But being shouted at, that pained me a lot. That is why I don’t come here I had even told them that I should be removed from the list. (31-year-old female, unknown partner HIV status)*

## Discussion

In this mixed methods study exploring the reasons for oral PrEP discontinuation among individuals in Kenya, our findings revealed that a majority of clients mainly discontinued PrEP for reasons aligned with low-HIV risk states. Specifically, more than three quarters of clients no longer felt at risk for HIV which led to PrEP discontinuation, which is consistent with the thematic findings from our qualitative data. Factors related to PrEP medication (e.g., side effects and pill burden) and opportunity costs (e.g., travel and waiting time) were reported less frequently.

Individual and interpersonal factors emerged as the main reasons for PrEP discontinuation among qualitative interview participants, particularly related to perceived low HIV risk including changes in relationship dynamics (either separation or partner relocation), partner viral suppression, and changes in behaviors associated with HIV risk, mainly reduced sexual activity. Similarly in our quantitative findings, the majority of participants discontinued PrEP because they no longer felt at HIV risk. These findings are comparable to existing literature where different populations in research settings—such as gay and bisexual men [32], the general population [18] and adolescent girls and young women [33]—have discontinued PrEP due to low HIV risk perception [19]. Our study also delves deeper into understanding how individuals perceive and assess low HIV risk, suggesting that participants were able to reasonably assess the period within which they were at a high risk of HIV acquisition (i.e., ‘seasons of risks’), [34,35]. Notably, some participants demonstrated an understanding of the concept undetectable = untransmittable, and reported that their partners’ viral suppression motivated them to discontinue PrEP, as they felt sufficiently protected against HIV. This suggests that counseling on PrEP adherence aligned with HIV risk may ensure accurate self-assessment and timing of seasons of HIV risk, to ensure correct PrEP use [23,36]. Prior research has demonstrated that PrEP discontinuation during seasons of low HIV risk is appropriate; our findings further indicate that participants made informed decisions to discontinue PrEP during such seasons, thus illustrating prevention-effective adherence knowledge [23].

Other individual and interpersonal factors that contributed to PrEP discontinuation among a few participants include pill burden, and experienced and anticipated side effects, which complement previous studies [18,32]. For example, a study conducted in the United States reported similar findings; majority of participants discontinued PrEP due to perceived low risk but only a few (~4%) discontinued as a result of experienced/anticipated side effects and pill burden [32]. Notably, this was a longitudinal cohort study among gay and bisexual men whereas our study was based in a real-world setting among the general population, demonstrating similar PrEP experiences among different populations in different contexts. PrEP initiation counselling could emphasize on expected side effects and duration, side effect management, and strategies to overcome adherence challenges [18,37].

Community-level factors such as perceived HIV and PrEP stigma also contributed to PrEP discontinuation among participants. Stigma has been shown to limit PrEP continuation among adolescent girls and young women in trials and PrEP users in HIV clinics [20,38]. In our study, participants described concerns about stigma related to frequenting and queuing at HIV clinics for PrEP and being perceived as living with HIV. Demedicalized PrEP delivery models, such as community-based pharmacy delivery [39] and integration with other routine care clinics (e.g., family planning clinics) [40], could help minimize stigma. PrEP awareness campaigns could also consider messaging that focuses on PrEP as an option for HIV prevention, rather than on individuals’ HIV risk [41].

We also found important structural factors, such as distance to HIV clinics and time constraints, contributed to PrEP discontinuation, which have been reported as a concern for PrEP users such as young men who have sex with men and the general public in other settings [18,42]. Our study however also revealed that long waiting times in HIV clinics and frequency of PrEP refill visits contributed to PrEP discontinuation. Various strategies to navigate logistical barriers associated with antiretroviral therapy delivery have been proven successful such as multi-month dispensing, fast-tracked refills and longer visit intervals [43,44]. Similarly, a trial conducted in Kenya found that semiannual PrEP dispensing supported with interim HIV self-testing reduced the frequency of clinic visits without compromising PrEP continuation outcomes, and was comparable to the standard of care [45]. These differentiated models of delivery have the potential to improve PrEP continuation in different settings and populations while addressing logistical barriers.

Our study had strengths and limitations. An important strength was the utilization of a mixed-methods design which enabled the triangulation of data from quantitative and qualitative methods, allowing an in-depth understanding of the reasons for PrEP discontinuation (S5 Appendix). While the quantitative data provided a broad perspective on the reasons for PrEP discontinuation, the qualitative data offered detailed insight into the individual, interpersonal, community and structural/institutional factors influencing these decisions. For example, where the quantitative data found that most participants discontinued PrEP due to low HIV risk perception or separation, the qualitative data elaborated this, and revealed that relationship dynamics such as separation, partner relocation, and viral suppression were considered low risk HIV states and led to discontinuation. Another strength is that participants were clients who received PrEP as part of standard of care at the selected public HIV clinics, giving insight into a real-world PrEP implementation setting. A limitation of this study is data were only collected among reachable participants; therefore, we did not gather the perspectives of those who were not reachable or who declined participation in the study. Future studies should explore innovative strategies such as community outreach programs and telehealth services [46], to reach populations that do not return for their clinic visits to understand their reasons for discontinuation. Reasons for PrEP discontinuation may also differ among different populations in other health system contexts, limiting the generalizability of our data. Majority of the participants in this study were from the general population or in serodiscordant partnerships, which may not represent the experiences of other individuals who discontinue PrEP such as pregnant and breastfeeding women. To gain deeper insight into PrEP discontinuation patterns, future research could engage diverse populations to capture their experiences. Another limitation of this study was conducting interviews over the phone, which limited interviewers’ ability to observe participants’ non-verbal cues, and ensure their attention throughout the interviews. To mitigate this, interviewers emphasized the importance of participants’ responses in understanding the research questions. Interviewers also used active listening techniques to encourage participants to continue speaking, and probing skills to elicit more information.

## Conclusion

To a large extent, individuals using PrEP were able to accurately assess their seasons of risk before making decisions to discontinue PrEP use. These findings reinforce the importance of national PrEP programs including training of healthcare providers on prevention-effective adherence counselling, to empower PrEP users to ensure this concept is correctly applied for optimal protection during periods of high HIV risk. Training of healthcare providers would also make it easy for end users to stop and restart PrEP, to align with their HIV risk profile.

## Supporting information

S1 Appendix Questionnaires. (PDF)

S2 Appendix In-depth interview guide. (PDF)

S3 Appendix Oral consent guide. (PDF)

S4 Appendix Oral consent log. (PDF)

S5 Appendix Joint display table of qualitative and quantitative findings. (PDF)

S1 Checklist PLOS questionnaire on inclusivity in global research. (PDF)

## References

[1] Thigpen MC, Kebaabetswe PM, Paxton LA, Smith DK, Rose CE, Segolodi TM, et al. Antiretroviral preexposure prophylaxis for heterosexual HIV transmission in Botswana. N Engl J Med. 2012;367(5):423–34. doi: 10.1056/NEJMoa1110711 22784038

[2] Baeten JM, Donnell D, Ndase P, Mugo NR, Campbell JD, Wangisi J, et al. Antiretroviral prophylaxis for HIV prevention in heterosexual men and women. N Engl J Med. 2012;367(5):399–410. doi: 10.1056/NEJMoa1108524 22784037 PMC3770474

[3] Haberer JE, Baeten JM, Campbell J, Wangisi J, Katabira E, Ronald A, et al. Adherence to antiretroviral prophylaxis for HIV prevention: a substudy cohort within a clinical trial of serodiscordant couples in East Africa. PLoS Med. 2013;10(9):e1001511. doi: 10.1371/journal.pmed.1001511 24058300 PMC3769210

[4] Stankevitz K, Grant H, Lloyd J, Gomez GB, Kripke K, Torjesen K, et al. Oral preexposure prophylaxis continuation, measurement and reporting. AIDS. 2020;34(12):1801–11. doi: 10.1097/QAD.0000000000002598 32558660 PMC8635251

[5] Zhang J, Li C, Xu J, Hu Z, Rutstein SE, Tucker JD, et al. Discontinuation, suboptimal adherence, and reinitiation of oral HIV pre-exposure prophylaxis: a global systematic review and meta-analysis. Lancet HIV. 2022;9(4):e254–68. doi: 10.1016/S2352-3018(22)00030-3 35364026 PMC9124596

[6] Celum C, Hosek S, Tsholwana M, Kassim S, Mukaka S, Dye BJ, et al. PrEP uptake, persistence, adherence, and effect of retrospective drug level feedback on PrEP adherence among young women in southern Africa: Results from HPTN 082, a randomized controlled trial. PLoS Med. 2021;18(6):e1003670. doi: 10.1371/journal.pmed.1003670 34143779 PMC8253429

[7] Celum CL, Delany-Moretlwe S, Baeten JM, van der Straten A, Hosek S, Bukusi EA, et al. HIV pre-exposure prophylaxis for adolescent girls and young women in Africa: from efficacy trials to delivery. J Int AIDS Soc. 2019;22:e25298. doi: 10.1002/jia2.25298 31328444 PMC6643076

[8] de Dieu Tapsoba J, Zangeneh SZ, Appelmans E, Pasalar S, Mori K, Peng L, et al. Persistence of oral pre-exposure prophylaxis (PrEP) among adolescent girls and young women initiating PrEP for HIV prevention in Kenya. AIDS Care. 2021;33(6):712–20. doi: 10.1080/09540121.2020.1822505 32951437 PMC7981281

[9] Mugwanya KK, Palayew A, Schaafsma T, Irungu EM, Bukusi E, Mugo N, et al. Patterns of PrEP continuation and coverage in the first year of use: a latent class analysis of a programmatic PrEP trial in Kenya. J Int AIDS Soc. 2023;26(7):e26137. doi: 10.1002/jia2.26137 37403405 PMC10320042

[10] Odoyo JB, Morton JF, Ngure K, O’Malley G, Mugwanya KK, Irungu E, et al. Integrating PrEP into HIV care clinics could improve partner testing services and reinforce mutual support among couples: provider views from a PrEP implementation project in Kenya. J Int AIDS Soc. 2019;22:e25303. doi: 10.1002/jia2.25303 31321911 PMC6639665

[11] Baeten JM, Heffron R, Kidoguchi L, Mugo NR, Katabira E, Bukusi EA, et al. Integrated Delivery of Antiretroviral Treatment and Pre-exposure Prophylaxis to HIV-1-Serodiscordant Couples: A Prospective Implementation Study in Kenya and Uganda. PLoS Med. 2016;13(8):e1002099. doi: 10.1371/journal.pmed.1002099 27552090 PMC4995047

[12] Irungu EM, Mugwanya KK, Mugo NR, Bukusi EA, Donnell D, Odoyo J, et al. Integration of pre-exposure prophylaxis services into public HIV care clinics in Kenya: a pragmatic stepped-wedge randomised trial. Lancet Glob Health. 2021;9(12):e1730–9. doi: 10.1016/S2214-109X(21)00391-0 34798031 PMC8609282

[13] Masyuko S, Mukui I, Njathi O, Kimani M, Oluoch P, Wamicwe J, et al. Pre-exposure prophylaxis rollout in a national public sector program: the Kenyan case study. Sex Health. 2018;15(6):578–86. doi: 10.1071/SH18090 30408432 PMC7206896

[14] National AIDS & STI Control Programme (NASCOP). Ministry of Health. Framework for the Implementation of Pre-exposure Prophylaxis of HIV in Kenya. Nairobi, Kenya: NASCOP; 2022.

[15] Irungu EM, Odoyo J, Wamoni E, Bukusi EA, Mugo NR, Ngure K, et al. Process evaluation of PrEP implementation in Kenya: adaptation of practices and contextual modifications in public HIV care clinics. J Int AIDS Soc. 2021;24(9):e25799. doi: 10.1002/jia2.25799 34496148 PMC8425783

[16] Omollo V, Roche SD, Mogaka F, Odoyo J, Barnabee G, Bukusi EA, et al. Provider-client rapport in pre-exposure prophylaxis delivery: a qualitative analysis of provider and client experiences of an implementation science project in Kenya. Sex Reprod Health Matters. 2022;30(1):2095707. doi: 10.1080/26410397.2022.2095707 36169648 PMC9542727

[17] Kinuthia J, Pintye J, Abuna F, Mugwanya KK, Lagat H, Onyango D, et al. Pre-exposure prophylaxis uptake and early continuation among pregnant and post-partum women within maternal and child health clinics in Kenya: results from an implementation programme. Lancet HIV. 2020;7(1):e38–48. doi: 10.1016/S2352-3018(19)30335-2 31813837 PMC11498332

[18] Bärnighausen K, Geldsetzer P, Matse S, Hettema A, Hughey AB, Dlamini P, et al. Qualitative accounts of PrEP discontinuation from the general population in Eswatini. Cult Health Sex. 2021;23(9):1198–214. doi: 10.1080/13691058.2020.1770333 32633617

[19] Rousseau E, Katz AWK, O’Rourke S, Bekker L-G, Delany-Moretlwe S, Bukusi E, et al. Adolescent girls and young women’s PrEP-user journey during an implementation science study in South Africa and Kenya. PLoS One. 2021;16(10):e0258542. doi: 10.1371/journal.pone.0258542 34648589 PMC8516266

[20] Ongolly FK, Dolla A, Ngure K, Irungu EM, Odoyo J, Wamoni E, et al. “I Just Decided to Stop:” Understanding PrEP Discontinuation Among Individuals Initiating PrEP in HIV Care Centers in Kenya. J Acquir Immune Defic Syndr. 2021;87(1):e150–8. doi: 10.1097/QAI.0000000000002625 33492024 PMC8026512

[21] Ngure K, Thuo N, Ogello V, Kiptinness C, Kamolloh K, Burns BFO, et al. Dynamic Perceived HIV Risk and Sexual Behaviors Among Young Women Enrolled in a PrEP Trial in Kenya: A Qualitative Study. Front Reprod Health. 2021;3:637869. doi: 10.3389/frph.2021.637869 36304002 PMC9580724

[22] Irungu EM, Baeten JM. PrEP rollout in Africa: status and opportunity. Nat Med. 2020;26(5):655–64. doi: 10.1038/s41591-020-0872-x 32405065

[23] Haberer JE, Bangsberg DR, Baeten JM, Curran K, Koechlin F, Amico KR, et al. Defining success with HIV pre-exposure prophylaxis: a prevention-effective adherence paradigm. AIDS. 2015;29(11):1277–85. doi: 10.1097/QAD.0000000000000647 26103095 PMC4480436

[24] The path that ends AIDS: UNAIDS Global AIDS Update 2023. Geneva: Joint United Nations Programme on HIV/AIDS; 2023. Licence: CC BY-NC-SA 3.0 IGO.

[25] Zewdie KB, Ngure K, Mwangi M, Mwangi D, Maina S, Etyang L, et al. Effect of differentiated direct-to-pharmacy PrEP refill visits; supported with client HIV self-testing on clinic visit time and early PrEP continuation. J Int AIDS Soc. 2024;27(3):e26222. doi: 10.1002/jia2.26222 38446643 PMC10935714

[26] Harris PA, Taylor R, Thielke R, Payne J, Gonzalez N, Conde JG. Research electronic data capture (REDCap)--a metadata-driven methodology and workflow process for providing translational research informatics support. J Biomed Inform. 2009;42(2):377–81. doi: 10.1016/j.jbi.2008.08.010 18929686 PMC2700030

[27] Patton MQ. Qualitative research & evaluation methods. 3rd ed. Thousand Oaks, CA: Sage Publications; 2002.

[28] Pope C, Mays N. Analysing Qualitative Data. Qualitative Research in Health Care. Blackwell Publishing Ltd; 2006. p. 63–81.

[29] Diez Roux AV. The study of group-level factors in epidemiology: rethinking variables, study designs, and analytical approaches. Epidemiol Rev. 2004;26:104–11. doi: 10.1093/epirev/mxh006 15234951

[30] Feldacker C, Ennett ST, Speizer I. It’s not just who you are but where you live: an exploration of community influences on individual HIV status in rural Malawi. Soc Sci Med. 2011;72(5):717–25. doi: 10.1016/j.socscimed.2011.01.003 21316134

[31] Tong A, Sainsbury P, Craig J. Consolidated criteria for reporting qualitative research (COREQ): a 32-item checklist for interviews and focus groups. Int J Qual Health Care. 2007;19(6):349–57. doi: 10.1093/intqhc/mzm042 17872937

[32] Whitfield THF, John SA, Rendina HJ, Grov C, Parsons JT. Why I Quit Pre-Exposure Prophylaxis (PrEP)? A Mixed-Method Study Exploring Reasons for PrEP Discontinuation and Potential Re-initiation Among Gay and Bisexual Men. AIDS Behav. 2018;22(11):3566–75. doi: 10.1007/s10461-018-2045-1 29404756 PMC6077114

[33] Ohiomoba RO, Owuor PM, Orero W, Were I, Sawo F, Ezema A, et al. Pre-Exposure Prophylaxis (PrEP) Initiation and Retention Among Young Kenyan Women. AIDS Behav. 2022;26(7):2376–86. doi: 10.1007/s10461-022-03576-x 35061115

[34] Haberer JE. Current concepts for PrEP adherence in the PrEP revolution: from clinical trials to routine practice. Curr Opin HIV AIDS. 2016;11(1):10–7. doi: 10.1097/COH.0000000000000220 26633638 PMC4801217

[35] Mugo NR, Ngure K, Kiragu M, Irungu E, Kilonzo N. The preexposure prophylaxis revolution; from clinical trials to programmatic implementation. Curr Opin HIV AIDS. 2016;11(1):80–6. doi: 10.1097/COH.0000000000000224 26575147 PMC4900687

[36] Namey E, Agot K, Ahmed K, Odhiambo J, Skhosana J, Guest G, et al. When and why women might suspend PrEP use according to perceived seasons of risk: implications for PrEP-specific risk-reduction counselling. Cult Health Sex. 2016;18(9):1081–91. doi: 10.1080/13691058.2016.1164899 27093238 PMC5049692

[37] Van der Elst EM, Mbogua J, Operario D, Mutua G, Kuo C, Mugo P, et al. High acceptability of HIV pre-exposure prophylaxis but challenges in adherence and use: qualitative insights from a phase I trial of intermittent and daily PrEP in at-risk populations in Kenya. AIDS Behav. 2013;17(6):2162–72. doi: 10.1007/s10461-012-0317-8 23080358 PMC3690654

[38] Velloza J, Khoza N, Scorgie F, Chitukuta M, Mutero P, Mutiti K, et al. The influence of HIV-related stigma on PrEP disclosure and adherence among adolescent girls and young women in HPTN 082: a qualitative study. J Int AIDS Soc. 2020;23(3):e25463. doi: 10.1002/jia2.25463 32144874 PMC7060297

[39] Ortblad KF, Mogere P, Omollo V, Kuo AP, Asewe M, Gakuo S, et al. Stand-alone model for delivery of oral HIV pre-exposure prophylaxis in Kenya: a single-arm, prospective pilot evaluation. J Int AIDS Soc. 2023;26(6):e26131. doi: 10.1002/jia2.26131 37306128 PMC10258863

[40] Mugwanya KK, Pintye J, Kinuthia J, Abuna F, Lagat H, Begnel ER, et al. Integrating preexposure prophylaxis delivery in routine family planning clinics: A feasibility programmatic evaluation in Kenya. PLoS Med. 2019;16(9):e1002885. doi: 10.1371/journal.pmed.1002885 31479452 PMC6719826

[41] Rivet Amico K, Bekker L-G. Global PrEP roll-out: recommendations for programmatic success. Lancet HIV. 2019;6(2):e137–40. doi: 10.1016/S2352-3018(19)30002-5 30660592

[42] Morgan E, Ryan DT, Newcomb ME, Mustanski B. High Rate of Discontinuation May Diminish PrEP Coverage Among Young Men Who Have Sex with Men. AIDS Behav. 2018;22(11):3645–8. doi: 10.1007/s10461-018-2125-2 29728950 PMC6204096

[43] Prust ML, Banda CK, Nyirenda R, Chimbwandira F, Kalua T, Jahn A, et al. Multi-month prescriptions, fast-track refills, and community ART groups: results from a process evaluation in Malawi on using differentiated models of care to achieve national HIV treatment goals. J Int AIDS Soc. 2017;20(Suppl 4):21650. doi: 10.7448/IAS.20.5.21650 28770594 PMC5577715

[44] Mody A, Roy M, Sikombe K, Savory T, Holmes C, Bolton-Moore C, et al. Improved Retention With 6-Month Clinic Return Intervals for Stable Human Immunodeficiency Virus-Infected Patients in Zambia. Clin Infect Dis. 2018;66(2):237–43. doi: 10.1093/cid/cix756 29020295 PMC5850531

[45] Ortblad KF, Bardon AR, Mogere P, Kiptinness C, Gakuo S, Mbaire S, et al. Effect of 6-Month HIV Preexposure Prophylaxis Dispensing With Interim Self-testing on Preexposure Prophylaxis Continuation at 12 Months: A Randomized Noninferiority Trial. JAMA Netw Open. 2023;6(6):e2318590. doi: 10.1001/jamanetworkopen.2023.18590 37318803 PMC10273023

[46] Henderson M, Schmidt H-MA, Chitembo L, Peralta H, Alaama AS, Johnson C, et al. The Future of Pre-Exposure Prophylaxis (PrEP) for HIV Prevention: A Global Qualitative Consultation on Provider Perspectives on New Products and Differentiated Service Delivery. AIDS Behav. 2023;27(11):3755–66. doi: 10.1007/s10461-023-04093-1 37351685 PMC10589125

