# Supplementary material for: Low HIV-risk aligned discontinuation among HIV pre-exposure prophylaxis users within public HIV clinics in Kenya: A mixed method study
Source: PLOS Glob Public Health. 2025 Apr 28;5(4):e0004493. doi: 10.1371/journal.pgph.0004493 (PMC12036852; doi:10.1371/journal.pgph.0004493)
Supplement: S1 Appendix — (PDF) [file pgph.0004493.s001.pdf]

Prep Discontinuation

Client PrEP ID	______________________
Date the interview was completed	______________________
Facility Name	◯ [redacted]<br>◯<br>◯<br>◯
Age	______________________
Sex at birth	◯ Female<br>◯ Male<br>(added 02/24/2022)
Participant's current marital status:	◯ Never married<br>◯ Cohabiting<br>◯ Married, monogamous<br>◯ Married, polygamous<br>◯ Separated/divorced<br>◯ Widowed
Participant's highest level of education:	◯ No schooling<br>◯ Primary school, not complete<br>◯ Primary school, complete<br>◯ Secondary school, not complete<br>◯ Secondary school, complete<br>◯ Attended post-secondary school<br>(added 12/12/2021)
What is the participant's primary job/occupation?	◯ Unemployed/no job<br>◯ Student<br>◯ Employment with a steady salary/formal sector (e.g. teaching, office work)<br>◯ Employment without a steady salary/informal sector (e.g. trader, selling goods)<br>◯ Parent caring for own child/housewife<br>◯ Household help/child care for others<br>◯ Casual work<br>◯ Other<br>(added 12/12/2021)

How much does the participant earn per month?

◯ No income
◯ 1 - 5,000 Ksh
◯ 5,001 - 10,000 Ksh
◯ > 10,000 Ksh
◯ Declines to answer
(added 12/12/2021)

CHARACTERISTICS AT PrEP INITIATION: ABSTRACT FROM PrEP CARD

Date of PrEP initiation:

____

Confirm calendar month of PrEP initiation

◯ January
◯ February
◯ March
◯ April
◯ May
◯ June
◯ July
◯ August
◯ September
◯ October
◯ November
◯ December

Marital status at PrEP initiation (select one):

◯ Single
◯ Cohabiting
◯ Married monogamous
◯ Married polygamous
◯ Separated/divorced
◯ Widowed

Previous PrEP use before coming to this clinic?

◯ Yes
◯ No
◯ Unknown-No entry

Population type (select one):

◯ Gen pop
◯ Key pop
◯ Fisher Folk
◯ Discordant Couple
◯ MSM
◯ PWID
◯ FSW
◯ Bridging pop
◯ Unknown-No entry

Referred from (select one):

◯ OPD
◯ VCT/HTS
◯ Straight to CCC
◯ MCH
◯ FP
◯ TB clinic
◯ IPD
◯ Peer
◯ Outreach
◯ Self-referral
◯ HBTC
◯ Other
◯ Unknown-No entry

Behaviour risk assessment (check all that apply):	◯ Sex partner is HIV+<br>◯ Sex partner is at high risk for HIV and HIV status is unknown<br>◯ Has sex with >1 partner<br>◯ Ongoing IPV/GBV<br>◯ Transactional sex<br>◯ Recent STI<br>◯ Recent use of PEP<br>◯ Recurrent sex under influence of alcohol or recreational drugs<br>◯ Inconsistent or no condom use<br>◯ Injection drug use with shared needles and/or syringes<br>◯ Unknown-No entry
HIV+ partner on ART at initial visit:	◯ Yes<br>◯ No<br>◯ Unknown-no entry
Number of months since HIV+ partner started ART:	◯ < 6 months<br>◯ 6-12 months<br>◯ >12 months (>1 yr)<br>◯ Not known<br>◯ Unknown-no entry
Sex without a condom with HIV+ partner in past 30 days:	◯ Yes<br>◯ No<br>◯ Unknown-no entry
Number of living children with HIV+ partner	______________________________
Male only-circumcised	◯ Yes<br>◯ No<br>◯ Unknown-no entry
Pregnant or Breastfeeding?	◯ Not pregnant or breastfeeding<br>◯ pregnant<br>◯ Breastfeeding<br>◯ Unknown-no entry
FP methods use:	◯ None<br>◯ Condoms<br>◯ Implant<br>◯ Fertility awareness<br>◯ Emergency contraceptives<br>◯ Intrauterine device<br>◯ Tubal ligation<br>◯ Oral contraceptives<br>◯ Lactational amenorrhea<br>◯ Hysterectomy<br>◯ Injectable<br>◯ Diaphragm/cervical cap<br>◯ MEN - post-menopausal<br>◯ Unknown-No entry
Plan to have children (select one):	◯ Trying to conceive<br>◯ Future<br>◯ Don't know<br>◯ No

Fears about starting PrEP (mark all that apply):

◯ None
◯ Side effects (ADR)
◯ Stigma
◯ Pill burden
◯ Taking pills for a long time
◯ Other
◯ Unknown-No entry

END OF ABSTRACT FROM PrEP CARD

When did you last test for HIV?

◯ =< 1 month
◯ 2-3 months
◯ 4- 6 months
◯ 7-12 month
◯ >12 months

What was the HIV results of that test.

◯ Negative
◯ Postive
◯ Indeterminate
◯ Not sure

When is the last time you took PrEP

◯ =< 1 month
◯ 2-3 months
◯ 4- 6 months
◯ 7-12 month
◯ >12 months

Are you currently on PrEP?

◯ Yes
◯ No

For how many months did you take PrEP?

◯ =< 1 month
◯ 2-3 months
◯ 4- 6 months
◯ 7-12 month
◯ >12 months

What was the main reason you stopped taking PrEP?

◯ Clinician initiated PrEP stop
◯ Did not have enough information about using PrEP
◯ Felt I was no longer at risk for HIV
◯ Concerned about too many pills to take every day
◯ PrEP is not the right HIV prevention method for me
◯ Concerned about family finding out I am taking PrEP
◯ Concerned about friends/others finding out I am taking PrEP
◯ Concerned that PrEP is not effective
◯ Did not trust the healthcare providers and the information given about PrEP
◯ Concerned about STIGMA
◯ Had or was concerned about side effects
◯ Did not know anyone else taking PrEP
◯ Concerned about too many visits
◯ Wait time at clinic was too long
◯ Concerned about transport to and from the clinic
◯ Concerned about missing work or losing work
◯ Other, specify:
◯ HIV+ partner on ART and virally suppressed
◯ Stopped after Conceiving
◯ separated-- no longer at risk

What are the other reasons that contributed to you stopping PrEP or not returning for your PreP visits the clinics	☐ Clinician initiated PrEP stop<br>☐ Did not have enough information about using PrEP<br>☐ Felt I was no longer at risk for HIV<br>☐ Concerned about too many pills to take every day<br>☐ PrEP is not the right HIV prevention method for me<br>☐ Concerned about family finding out I am taking PrEP<br>☐ Concerned about friends/others finding out I am taking PrEP<br>☐ Concerned that PrEP is not effective<br>☐ Did not trust the healthcare providers and the information given about PrEP<br>☐ Had or was concerned about side effects<br>☐ Did not know anyone else taking PrEP<br>☐ Concerned about too many visits<br>☐ Wait time at clinic was too long<br>☐ Concerned about transport to and from the clinic<br>☐ Concerned about missing work or losing work<br>☐ Stigma<br>☐ Other, specify:<br>☐ HIV+ partner on ART and virally suppressed<br>☐ Stopped after Conceiving<br>☐ separated-- no longer at risk<br>(Added December 12, 2021)
At the time you started PrEP was it your decision to start PrEP or it is the health provider who decided that you start PrEP	◯ It was my own decision to start PrEP<br>◯ It was the healthcare provider decision that I start PrEP<br>◯ We agreed together with the provider that I start PrEP<br>(added 12/12/2021)
At the time you initiated PrEP, how did you feel about your chance of getting infected with HIV?	◯ Very high chance<br>◯ Some chance<br>◯ Neither high nor low<br>◯ Not chance at all

Now I would like to ask you a series of questions about things that may have influenced your decision to stop taking PrEP.
For each item, please let me know if how strongly it influenced your decision: Very strong influence, Strong influence, Little influence, or No influence at all

Influence on decision to stop: I felt I was no longer at risk for HIV	◯ Very strong influence<br>◯ Strong influence<br>◯ Little influence<br>◯ No influence at all
Did you feel you had enough or adequate information from the staff about using PrEP or not?	◯ Enough information<br>◯ Somewhat enough information<br>◯ Neutral<br>◯ Somewhat not enough information<br>◯ Not enough information at all
Influence on decision to stop: I felt I did not have enough information about using PrEP or not	◯ Very strong influence<br>◯ Strong influence<br>◯ Little influence<br>◯ No influence at all

Influence on decision to stop: I was concerned about taking pills every day.	◯ Very strong influence<br>◯ Strong influence<br>◯ Little influence<br>◯ No influence at all
Influence on decision to stop: PrEP is not the right HIV prevention option for me	◯ Very strong influence<br>◯ Strong influence<br>◯ Little influence<br>◯ No influence at all
Influence on decision to stop: I was concerned about family members finding out that I was using PrEP.	◯ Very strong influence<br>◯ Strong influence<br>◯ Little influence<br>◯ No influence at all
Influence on decision to stop: I was concerned about friends or other community member finding out that I was using PrEP.	◯ Very strong influence<br>◯ Strong influence<br>◯ Little influence<br>◯ No influence at all
Influence on decision to stop: I do not trust the healthcare providers and the information they give about PrEP.	◯ Very strong influence<br>◯ Strong influence<br>◯ Little influence<br>◯ No influence at all
Influence on decision to stop: I had concern about side effects	◯ Very strong influence<br>◯ Strong influence<br>◯ Little influence<br>◯ No influence at all
Influence on decision to stop: I didn't know anyone else taking PrEP	◯ Very strong influence<br>◯ Strong influence<br>◯ Little influence<br>◯ No influence at all
Influence on decision to stop: I was concerned that PrEP is not effective at preventing HIV.	◯ Very strong influence<br>◯ Strong influence<br>◯ Little influence<br>◯ No influence at all
How did you feel about number of clinic visits to get PrEP refill	◯ I had no concern with the frequency of visits at all<br>◯ Just about right<br>◯ Somewhat many visits<br>◯ Too many visits
Influence on decision to stop: I was concerned about too many visits to get PrEP	◯ Very strong influence<br>◯ Strong influence<br>◯ Little influence<br>◯ No influence at all
At my last clinic visit, I felt I waited too much, too little, or just the right amount of time	◯ Too much time<br>◯ A little more time<br>◯ Just the right amount of time

Influence on decision to stop: I felt the wait time at the clinic was too long.	◯ Very strong influence<br>◯ Strong influence<br>◯ Little influence<br>◯ No influence at all
Based on last visit at the clinic, how satisfied were you with your overall experience at the clinic?	◯ Totally dissatisfied<br>◯ Somewhat dissatisfied<br>◯ Somewhat satisfied<br>◯ Very satisfied<br>(added 12/12/2021)
Based on last visit at the clinic, how satisfied were you with how the clinic staff (i.e., healthcare providers) attitude or the way they treated you during your last visit to the clinic?	◯ Totally dissatisfied<br>◯ Somewhat dissatisfied<br>◯ Somewhat satisfied<br>◯ Very satisfied<br>(added 12/12/2021)
Influence on decision to stop: My experience with provider attitude and clinic services at my last visit	◯ Very strong influence<br>◯ Strong influence<br>◯ Little influence<br>◯ No influence at all
Influence on decision to stop: I was concerned about transport to and from the clinic	◯ Very strong influence<br>◯ Strong influence<br>◯ Little influence<br>◯ No influence at all
Influence on decision to stop: I was concerned about missing work/losing work.	◯ Very strong influence<br>◯ Strong influence<br>◯ Little influence<br>◯ No influence at all
In your opinion, how would you assess your chance of getting infected with HIV in the next three months?	◯ Very high chance<br>◯ Some chance<br>◯ Neither high nor low<br>◯ No chance at all
Do you feel your sexual partner's behavior gives you a chance of getting HIV in the next three months.	◯ Strongly agree<br>◯ Agree<br>◯ Disagree<br>◯ Strongly disagree<br>◯ no partner at the moment
In the past three months, which things or situations have made you think about whether you might get HIV?	◯ Nothing<br>◯ Being in a new relationship<br>◯ Partner traveling or out of town<br>◯ Drinking alcohol<br>◯ Feeling like my partner is hiding something from me<br>◯ Not using condoms with my partner<br>◯ Feeling forced to have sex<br>◯ Hearing that someone I know has HIV<br>◯ Learning that a partner has HIV<br>◯ Not trusting or losing trust in my partner<br>◯ Casual partners<br>◯ Other<br>(added 12/12/2021)

How likely are you to restart using PrEP	◯ Very likely<br>◯ likely<br>◯ Neutral<br>◯ not likely<br>◯ Not likely at all
How likely are you to recommend other people to use PrEP for HIV prevention	◯ Very likely<br>◯ likely<br>◯ Neutral<br>◯ not likely<br>◯ Not likely at all
If you decided to restart PrEP, where (place) would you prefer to get your PrEP from	☐ HIV clinics<br>☐ Outpatient department<br>☐ Community pharmacy/drug shops<br>☐ Direct from pharmacy within health facilities<br>☐ Family planning clinics<br>☐ Antenatal clinics<br>☐ Private clinics<br>☐ Drop in centers<br>☐ STI clinics<br>☐ Mobile clinics<br>☐ Online<br>☐ Via phone or video chat<br>☐ Other
In general, where do you prefer to receive your HIV prevention services?	☐ HIV clinics<br>☐ Outpatient department<br>☐ Community pharmacy/drug shops<br>☐ Direct from pharmacy within health facilities<br>☐ Family planning clinics<br>☐ Antenatal clinics<br>☐ Private clinics<br>☐ Drop in centers<br>☐ STI clinics<br>☐ Mobile clinics<br>☐ Online<br>☐ Via phone or video chat<br>☐ Other<br>(added 12/12/2021)
Please tell me why you prefer to get your HIV prevention services there?	______________________________<br>(added 03/25/2025)
What are you currently using to protect yourself from getting HIV?	☐ None<br>☐ Consistent condom use<br>☐ My HIV+ partner is on ART<br>☐ Oral PrEP<br>☐ Abstinence<br>☐ I don't feel at risk for HIV<br>☐ I don't have a partner at the moment<br>☐ Other, specify<br>(Added 12/12/2021)

How satisfied are you with your current options for protecting yourself against HIV?	◯ Very satisfied<br>◯ Somewhat satisfied<br>◯ Neutral<br>◯ Somewhat dissatisfied<br>◯ Totally dissatisfied<br>(added 12/12/2021)

Now I would like to discuss about future new HIV prevention options.
Recently research showed that another form of PrEP using injectable given every two months works very well in preventing HIV infection in men and women.

Have you heard of injectable PrEP before today?	◯ Yes<br>◯ No<br>(added 12/12/2021)

For the next five questions please tell me how strongly you agree or disagree with the statement about injectable PrEP

I am interested in learning more about injectable PrEP for HIV prevention.	◯ Strongly agree<br>◯ Agree<br>◯ Disagree<br>◯ Strongly disagree<br>(added 12/12/2021)
I would consider using injectable PrEP to protect myself against HIV.	◯ Strongly agree<br>◯ Agree<br>◯ Disagree<br>◯ Strongly disagree<br>(added 12/12/2021)
If you are using oral PrEP would you consider switching to injectable PrEP for HIV prevention if it become available to you?	◯ Yes<br>◯ No<br>◯ Don't know<br>(added 12/12/2021)
What concern or fears, if any, do you have about injectable PrEP for HIV prevention?	☐ No concern/fears at all<br>☐ Safety or side effects<br>☐ Pain from the injections<br>☐ Having to return for injections every two months<br>☐ Fear/dislike of needles<br>☐ I do not have enough information about injectable PrEP<br>☐ No longer interested in PrEP<br>☐ Weight gain<br>☐ Other<br>(added 12/12/2021)
In the future, there could be many different types of HIV prevention methods available, like daily PrEP pill, a monthly PrEP pill, 2-monthly injectable PrEP, 6-monthly injectable PrEP, an implant, or a intravaginal ring.<br><br>Which type of PrEP would you prefer to use if they were available?	☐ Daily pill<br>☐ Monthly pill<br>☐ Intravaginal ring<br>☐ 2-monthly Injectable<br>☐ 6-monthly Injectable<br>☐ Implant<br>☐ No longer interested in PrEP<br>☐ Other<br>(added 12/12/2021)

What are your reasons for preferring the PrEP option mentioned above	______________________________<br>(added 03/09/2022)

SEXUAL BEHAVIOR

Now I would like to ask you about your sexual life

Including your primary partner, if you have one, how many total sex partners have you had in the past three months?	◯ Zero (0) partner<br>◯ One partner<br>◯ Two partners<br>◯ Three partners<br>◯ Four partners<br>◯ >= Five partners<br>(added 12/12/2021)
How many would you consider as main partners?	◯ Zero (0) partner<br>◯ One partner<br>◯ Two partners<br>◯ Three partners<br>◯ Four partners<br>◯ >= Five partners<br>(added 12/12/2021)
How many would you consider as casual partners?	◯ Zero (0) partner<br>◯ One partner<br>◯ Two partners<br>◯ Three partners<br>◯ Four partners<br>◯ >= Five partners<br>(added 12/12/2021)
In the past 3 months, how many times did you have vaginal sex with any partner?<br>By vaginal sex, we mean when a male puts his penis into your vagina.	______________________________<br>(added 12/12/2021)
Some people find it difficult to use condoms every time they have sex. In the past three months, how often was a condom used when you had vaginal sex?	◯ Always<br>◯ Often<br>◯ Sometimes<br>◯ Rarely<br>◯ Never
Some people find it difficult to use condoms every time they have sex. When you have sex with your main partner do you use condoms?	◯ Always<br>◯ Often<br>◯ Sometimes<br>◯ Rarely<br>◯ Never<br>(added 12/12/2021)
When you have sex with your casual partner (s) do you use condoms?	◯ Always<br>◯ Often<br>◯ Sometimes<br>◯ Rarely<br>◯ Never<br>(added 12/12/2021)

Was a condom used the most recent time you had vaginal sex?	◯ Yes<br>◯ No<br>◯ Part of the time<br>(added 12/12/2021)
What was the HIV status of the last person you had sex with?	◯ HIV negative<br>◯ HIV positive<br>◯ Unknown<br>(added 12/12/2021)

NASCOP HIV risk behavior characteristics

In the past six months: Have you had sex without a condom with a partner(s) of unknown or positive HIV status?	◯ Yes<br>◯ No<br>(added 12/12/2021)
In the past six months: Have you been diagnosed with or treated for an STI?	◯ Yes<br>◯ No<br>(added 12/12/2021)
In the past six months: Have you engaged in sex in exchange of money or other favors?	◯ Yes<br>◯ No<br>(added 12/12/2021)
In the past six months: Have you shared needles while engaging in intravenous drug use?	◯ Yes<br>◯ No<br>(added 12/12/2021)
In the past six months: Have you been forced to have sex against your will or physically assaulted, including assault by your sexual partner(s)?	◯ Yes<br>◯ No<br>(added 12/12/2021)
In the past six months: Have you used post-exposure prophylaxis (PEP) two times or more?	◯ Yes<br>◯ No<br>(added 12/12/2021)
In the past six months: Have engaged in sex under the influence of alcohol or recreation drugs	◯ Yes<br>◯ No<br>(added 12/12/2021)

STIGMA

Now I would like to ask you about general feelings people in your community may have about PrEP.

I am not referring to any specific person taking PrEP and would like to know what you think.

How concerned were you about stigma of receiving your PrEP services from an HIV clinic?	◯ Very concerned<br>◯ Somewhat concerned<br>◯ Somewhat not concerned<br>◯ Not concerned at all<br>(added 12/12/2021)
Some people in my community think people who take PrEP are promiscuous (have casual sex).	◯ Strongly agree<br>◯ Agree<br>◯ Disagree<br>◯ Strongly disagree<br>(added 12/12/2021)

People in my community think people who take PrEP are being responsible about their sexual health.	◯ Strongly agree<br>◯ Agree<br>◯ Disagree<br>◯ Strongly disagree<br>(added 12/12/2021)
People in my community think taking PrEP means you have HIV.	◯ Strongly agree<br>◯ Agree<br>◯ Disagree<br>◯ Strongly disagree<br>(Added 12/12/2021)
People in my community think PrEP may not be safe for your health.	◯ Strongly agree<br>◯ Agree<br>◯ Disagree<br>◯ Strongly disagree<br>(added 12/12/2021)

Now, I want discuss ways make access to prevention services better.

Use of mobile phone and video chats eg through whatApps could help clients to talk to staff without requiring you to come to the clinic. Also testing HIV by yourself either at home or within the facility could help reduce number of times you come to clinic or the waiting at the clinic

For the next four questions please tell me how strongly you agree or disagree with the statement about phone visits or HIV self testing for PrEP services

I would consider to have some of my PrEP services via phone or video chats on phone	◯ Strongly agree<br>◯ Agree<br>◯ Disagree<br>◯ Strongly disagree<br>(added 12/12/2021)
I would consider self testing for HIV at home as an option to have better PrEP services	◯ Strongly agree<br>◯ Agree<br>◯ Disagree<br>◯ Strongly disagree<br>(added 12/12/2021)
What concerns or fears if any do you have about phone visits or HIV self-testing for PrEP services	________________________________<br>(added 12/12/2021)
What advice can give on how you want to receive HIV prevention services including PrEP?<br><br>or In what ways should PrEP services be improved to meet your needs?	________________________________<br>(added 12/12/2021: Quote as closely as possible what the client says in their own words.)
STUDY STAFF SUMMARY NOTES	________________________________<br>(Added 12/12/2021)
Staff initials	________________________________
