# Supplementary material for: Low HIV-risk aligned discontinuation among HIV pre-exposure prophylaxis users within public HIV clinics in Kenya: A mixed method study
Source: PLOS Glob Public Health. 2025 Apr 28;5(4):e0004493. doi: 10.1371/journal.pgph.0004493 (PMC12036852; doi:10.1371/journal.pgph.0004493)
Supplement: S2 Appendix — (PDF) [file pgph.0004493.s002.pdf]

In-Depth Interview Guide
Protocol v2.0_10 October 2019

Instructions for interviewer:

1. Please write down if the interview is conducted during implementation of pharmacy refill or not.
2. Introduce yourself. Let the participant know why they were selected for the interview assure them that the information they share with you will be confidential and ensure that they have consented to the interview.

3. Primary interview questions are noted in **bold** text. As the interviewer, these are questions that must be asked or discussed with the participant. You don't have to read the questions verbatim, but are provided to ensure consistency across IDIs. The questions can be asked in any order depending on how the discussion with the participant is going, but by the end of the interview, all questions in bold should be asked.

4. Probing questions are indicated with a bullet. If a participant responds to the primary question with very little information, then these questions may be used to encourage further discussion. You do not have to cover all the probes provided, and you may not need to ask them if participant responds with enough information.

[Start recorder and read introduction]:

Instructions:
The following is a guide. Try to ask all the questions below in the order given, but it is more important to maintain the flow of discussion. Suggested probes have been included. Start with the following introductory script:
****
Hi, my name is_____________. Thank you for agreeing to participate in an interview today. I am interested in understanding your thoughts, experiences and opinions about doing the Pharmacy refill PrEP implementation in your facility. I will ask you questions that you are free to answer in any way you wish. Feel free to elaborate on any of your points. If a question is unclear to you, please feel free to ask me to explain it.

Before we start, I would like to remind you that there are no wrong answers in this discussion. We are interested in knowing what you think, so please feel free to be open and share your point of view. We hope you can help us understand what and does work in the PrEP/Pharmacy refill PrEP *so that we can make changes in the future. Your comments about what did not work are just as helpful as your comments about what did work. It is very important that we hear your opinion. You do not have to answer all the questions. If you want to stop the discussion at any time, just let me know.*
****

IDI guide for users who discontinue PrEP

Topic 1: Generation experience about PrEP

- What was your motivation to start PrEP?
- Tell me about your experience with PrEP use.
- Are you still taking PrEP?
    - If yes, where do get your PrEP from? What made you start taking PrEP there?
    - If no, when did you last take PrEP?
- Thinking back about the first time you were given PrEP; did you feel you were given enough information to enable you decide about using PrEP? What made you feel that way?
    - How adequate was the information on why you need to be on PrEP, possible side effects of PrEP, how often to use PrEP, how long you were going to use PrEP, and when you could stop PrEP?
- How did your experience on the first day influence your motivation to continue PrEP use?

Topic 2: Overall reasons for discontinuing PrEP.

- Please tell me about your decision to stop using PrEP?
- What was the main reason you decided to stop PrEP?
- What are the other reasons that influenced you to stop PrEP or coming to the clinic?

Topic 3: Last clinic visit experience

- Please tell me about your experience during your last clinic visit.
- Was that experience similar or different from other visits?
- How did this last visit influence your motivation to continue PrEP use?

Topic 4: Waiting time

- Thinking about your last visit at the clinic, what did you think of the time you spent there? (i.e., did you feel you waited for a long time, normal time or for a very short time)?
- How was that experience similar or different from other visits?
  How did the waiting time on your last visit influence your motivation to continue PrEP use?

Topic 5: Side effects

- What concerns or fears did/do you have about PrEP? If yes, what concerns?
- How did these concerns affect your motivation to continue PrEP use?

Topic 6: PrEP pill burden

- Please tell me about your experience with taking PrEP daily?
- Did you have concerns about having to take PrEP every day? What were the concerns?
- How did having to take PrEP every day affect your motivation to continue PrEP use?

Topic 7: Stigma and Social Norms:

- Please tell me how you felt about coming to an HIV clinic for PrEP.
- What concerns did you have about family members or other people finding you in an HIV clinic? If yes, what concerns? If no, why not?
- What concerns did you have about family members finding out that you are taking PrEP? If yes, what concerns?
- How did those concerns you mentioned affect your motivation to continue PrEP use?

Topic 8: Frequency of clinic visits:

- What do you think about the number of times/visits you had to come pick your PrEP?
- How did you feel about the frequency of visits?
- How did the frequency of return visits affect your motivation to continue PrEP use?

Topic 9: Provider attitudes:

- What was your experience with health providers at the clinic? Could you please give an example of a positive and a negative interaction with a provider at the clinic? If so, why?

Topic 10: Community awareness

- Please tell me what happened the first time you went back home with PrEP.
- Did you tell other people about PrEP? If yes, what was their reaction? How did their reaction influence your motivation to continue PrEP use?
- Do you know friends or other people in your community who are using PrEP? How did that influence your motivation to discontinue/continue PrEP use?

Topic 11: PrEP restarting

- Are you considering to restart PrEP? Why
- If you decided to restart PrEP, where would you want to get your PrEP from? Why?
- Would receiving PrEP at pharmacy/drug shop, family planning clinic be appealing to you? Why?

Topic 12: PrEP efficacy

- How well do you think PrEP works to prevent HIV? Please explain.
- How did that influence your motivation to discontinue/continue PrEP use?

Topic 13: PrEP formulations

- What do you think about using PrEP in other formulations than the oral pill (injection, implants, vaginal ring)?
- What dosage duration would you prefer (e.g., monthly, every 2 months, every 6 months, yearly, other)? Why?
- Which of these PrEP formulations would you personally prefer (1 month pill, 2 month injection, 6 month injection, 1 year implants, monthly vaginal ring)? Why?
- Which of these PrEP formulations do you think your peers would prefer? (1 month pill, 2 month injection, 6 month injection, implants, vaginal ring)? Why?
- How would receiving a different PrEP formulation influence your motivation to continue/ discontinue PrEP use?

Topic 14: PrEP and Family Planning (FP) - [*for female participants only*]

- Which family planning methods have you ever used? (Probe for 3-10 year IUCD, 5 year implant, 3 month injectable, daily oral pill).
- Which of the FP methods you've mentioned is your most preferred one? Why/ why not?
- Would you prefer to receive a PrEP formulation similar to your most preferred FP method? Why/ why not?
- What differences are there, if any, in your preference of FP method versus PrEP formulation?
- How would receiving a PrEP formulation similar to your preferred FP method influence your motivation to continue/ discontinue PrEP use?

Topic 15: HIV testing and prevention

- When was the last time you tested for HIV?
- Which HIV prevention methods are you currently using, if any? Why or why not?

Topic 16: General attitudes and beliefs:

- What does your community think about PrEP? How did that influence your motivation to continue PrEP use?
- Do you feel PrEP use should be encouraged? Why/Why not?
- Would you recommend other persons to consider PrEP use? Why do you feel that way?

Topic 17: Trust in health system and providers:

- Generally, do you believe the existing healthcare services in facilities are able to address your HIV prevention needs and preferences?
- Did you trust the information healthcare providers gave you about PrEP? What makes you feel that way?

Before we finish, could you share any other thoughts that you have about how PrEP services in general could be improved?

Possible probes:

- Is there anything we could change about waiting times, frequency of return visits, interactions with providers, improving privacy or reducing stigma, any other changes to help you/others continue PrEP use?

We have reached the end of our discussion. Thank you so much for your time and for sharing your opinions. Your contributions have been very helpful.
