# Supplementary material for: Low HIV-risk aligned discontinuation among HIV pre-exposure prophylaxis users within public HIV clinics in Kenya: A mixed method study
Source: PLOS Glob Public Health. 2025 Apr 28;5(4):e0004493. doi: 10.1371/journal.pgph.0004493 (PMC12036852; doi:10.1371/journal.pgph.0004493)
Supplement: S3 Appendix — (PDF) [file pgph.0004493.s003.pdf]

Approved
7/22/2021
UW IRB

Oral Consent Guide for users who discontinue PrEP

It is important to cover the following topics with the program participants:

Background information:

- Willingness to participate in this program is voluntary. You do not have to be in this study if you don't want to. You can withdraw at any time.
- Your usual care will not be affected by whether or not you participate in the study.
- KEMRI is coordinating this program to understand how to improve how to implement PrEP for HIV prevention as part of standard of care.

Investigators' statement: We are asking you to be part of research as part of efforts improve the national program to prevent HIV using PrEP medications. The purpose of this consent is to give you the information to help you decide if you want to take part. You may ask questions about:

- The purpose of the research
- What we would ask you to do
- The possible risks and benefits
- Your rights as a volunteer
- Anything else about the research or this form that is not clear

This process is called 'informed consent.'

Purpose of the Research: The goal of this program is to understand challenges which people who use PrEP face in order to help the Ministry of Health improve services for people who need or may benefit from PrEP as an HIV prevention strategy.

Program Procedure: If you decided to participate: We will ask you questions about your experience receiving and reasons for your decision to start and subsequently stop taking PrEP. We may also schedule your interview to take place remotely over the phone or internet. The interview/survey will take no more than two hours. We will record the interviews so we can write them down later. The audio recordings from the interviews will be destroyed by the end of the project in accordance with records retention requirements.

Risks, Stress or Discomfort: You may feel embarrassed when asked some question about your sexual life or experience with PrEP.

Benefits: There is no financial benefits for participating in this program but information you provide may help you and other to have better access to HIV prevention services

Other information:

- Your information will be kept private and any publication of this project will not use your name or identify you personally.
- This project is sponsored by the University of Washington and the National Institutes of Health, which are located in the USA in partnership with the Kenya Medical Research Institution.
- Offer to answer any questions they may have and give them clinic contact information for future questions.
- The information that we obtain from you for this study might be used for future studies. We may remove anything that might identify you from the information and specimens. If we do so, that information and specimens may then be used for future research studies or given to another investigator without getting additional permission from you. It is also possible that in the future we may want to use or share study information that might identify you. If we do, KEMRI Scientific and Ethics Review Unit and University Washington ethics board will decide whether or not we need to get additional permission from you.

Problems or questions

If you ever have any questions about this research, or if you have a research-related injury you should contact Dr. Elizabeth Irungu. If you have questions about your rights as a participant, contact the secretary of the KEMRI Scientific and Ethics Review Unit, P.O. Box 54840-00200, Nairobi, Telephone number 020272-2541, 0722205901, 0733-400003. Email address: seru@kemri.org.

Final Step:

- Ensure the participant understands what the program is about and if they have any questions
- Reemphasize that their data will be kept private and there will be no identifying links to participant.
- After you answer their questions verbally confirm that they agree to participant in the program today.

Sharing Test Results:

You will not have any tests done to you for this research. There will be no test results share.

Approved
7/22/2021
UW IRB
