# Supplementary material for: Low HIV-risk aligned discontinuation among HIV pre-exposure prophylaxis users within public HIV clinics in Kenya: A mixed method study
Source: PLOS Glob Public Health. 2025 Apr 28;5(4):e0004493. doi: 10.1371/journal.pgph.0004493 (PMC12036852; doi:10.1371/journal.pgph.0004493)
Supplement: S4 Appendix — (PDF) [file pgph.0004493.s004.pdf]

EFFICIENCY STUDY

PTID: ☐☐☐☐☐☐☐☐☐☐☐☐☐☐ **Date:** ☐☐ ☐☐☐ ☐☐

Participant ______________________________ agreed to participate in the discontinuation survey.

Name of person obtaining the consent: ______________________________

Signature of person obtaining the consent: ____________________ Date: ____________________
