# Supplementary material for: Low HIV-risk aligned discontinuation among HIV pre-exposure prophylaxis users within public HIV clinics in Kenya: A mixed method study
Source: PLOS Glob Public Health. 2025 Apr 28;5(4):e0004493. doi: 10.1371/journal.pgph.0004493 (PMC12036852; doi:10.1371/journal.pgph.0004493)
Supplement: S5 Appendix — (PDF) [file pgph.0004493.s005.pdf]

Joint Display Table of Qualitative and Quantitative Findings

Theme	Qualitative findings (n=30)	Quantitative findings (n=300)	Meta Inferences/Interpretation
Low HIV risk perception	Participants mainly discontinued PrEP when they perceived a low risk for HIV, which was influenced by factors such as separation from or relocation of their partners, partners achieving viral suppression or when healthcare providers advised them to stop PrEP use.	Similarly, 76% (229/300) discontinued PrEP due to perceived HIV low risk. Of these, 44% (131/300) generally felt they were no longer at risk, 23% (69/300) had separated from their partners, 6% (18/300) had partners with suppressed viral load and 3% (10/300) discontinued PrEP due to a healthcare provider's recommendation.	Both datasets demonstrate congruence, confirming that low HIV risk perception is a key reason for PrEP discontinuation. Qualitative findings provide specific contexts and nature of discontinuation that can help guide prevention-effective PrEP adherence.
Concerns about PrEP use	Participants discontinued PrEP after experiencing side effects (e.g., nausea, headache and general malaise) and reported concerns including fear of perceived side effects (e.g., cancer and kidney disease) from prolonged PrEP use and potential risks of taking PrEP alongside other medications.	PrEP use concerns were reported by 16% (47/300); 9% (26/300) were concerned about side effects and 6% (18/300) were concerned about pill burden.	There is congruence between data collected from surveys and in-depth interviews. Qualitative findings explain specific patient concerns of the nature of side effects that may be addressed through PrEP use counseling.

Logistical and accessibility challenges	Distance to HIV clinics, frequency of clinic visits, opportunity cost and facility challenges such as long queues, PrEP stockouts and negative provider attitude contributed to PrEP discontinuation.	Logistical barriers that led to PrEP discontinuation included accessibility concerns (4%;12/300), opportunity costs (1.3%;4/300) and long waiting time (0.3%;1/300).	Congruence between the two datasets.<br><br>Both findings point out logistical barriers to PrEP use. Qualitative findings provide specific client-level concerns and facility-level barriers that expose areas of improvement within facility set up.
HIV & PrEP stigma	HIV and PrEP stigma influenced PrEP discontinuation due to the fear of being seen at the HIV clinic and fear of inadvertent PrEP disclosure.	Only one participant (0.3%;1/300) discontinued PrEP due to concerns about others finding out.	The two datasets converge in identifying stigma as a reason for PrEP discontinuation. The qualitative data provides a more detailed description of participants' perceptions of stigma.<br>While only one participant reports stigma in the quantitative data, qualitative data highlights detailed description of how stigma in HIV clinics may impact PrEP use.
